# Curcumin’s effect on serum zinc, copper and magnesium levels in obese individuals

**DOI:** 10.22038/AJP.2022.21517

**Published:** 2023

**Authors:** Maryam Saberi-Karimian, Arezoo Orooji, Niloofar Taghizadeh, Mansoureh Sadat Ekhteraee Toosi, Gordon A. Ferns, Malihe Aghasizadeh, Majid Ghayour-Mobarhan

**Affiliations:** 1 *International UNESCO center for Health-Related Basic Sciences and Human Nutrition, Mashhad University of Medical Sciences, Mashhad, Iran*; 2 *Endoscopic and Minimally Invasive Surgery Research Center, Mashhad University of Medical Sciences, Mashhad, Iran*; 3 *Student Research Committee, Department of Epidemiology and Biostatistics, Faculty of Health, Mashhad University of Medical Sciences, Mashhad, Iran*; 4 *Student Research Committee, Faculty of Medicine, Mashhad University of Medical Sciences, Mashhad, Iran*; 5 *Brighton & Sussex Medical School, Division of Medical Education, Falmer, Brighton, Sussex BN1 9PH, UK*

**Keywords:** Obesity, Curcumin, Zinc, Copper, Magnesium

## Abstract

**Objective:**

The obesity prevalence is growing worldwide. There is strong evidence indicating that a disturbance of zinc, copper and magnesium concentrations is associated with the development of obesity and its related diseases. Our aim was to determine the effect of curcumin supplementation on serum zinc, magnesium and copper in obese individuals.

**Materials and Methods:**

In this randomized crossover trial study, thirty obese patients with an age range of 18 to 65 years were randomized to treatment with curcumin 1 g/day or placebo for 30 days. There was then a two-week wash-out period, after which, subjects crossed to the alternate regimen. Serum levels of zinc, copper and magnesium were determined at baseline and at the end of the study.

**Results:**

The study groups were similar to each other in base line characteristics. We did not observe significant impacts (p>0.05) of curcumin on Cu, Zn, Mg serum concentrations.

**Conclusion:**

Curcumin administration at a dose of 1 g/day for 30 days did not affect serum Cu, Zn, Mg levels in obese subjects.

## Introduction

During recent decades, obesity has been considered an important threat to public health (Organization, 2000). Obesity is defined as a body mass index *(*BMI)>30 kg/m^2^ and is associated with excessive accumulation of fat which may damage health (Pozza and Isidori, 2018). In addition, obesity is known as an independent risk factor for type 2 diabetes, dyslipidemia and cardiovascular diseases (CVDs) (Deedwania and Gupta, 2006). Due to poor nutrition in countries with obesity and overweight, lack of some micronutrients (zinc, copper, magnesium) and vitamins were reported (Norouzi et al., 2017). Studies show the important role of trace elements in human health. Copper and zinc are two essential elements in the diet that are involved in many biological reactions (Al-Fartusie and Mohssan, 2017). They act as cofactors in enzymatic reactions and show an essential role in various biochemical and metabolic processes in humans (Yatoo et al., 2013). Also, magnesium is the 4th most abundant cation in the body and acts as a cofactor (Guerrero-Romero and Rodriguez-Moran, 2002; Zocchi et al., 2021). Concerning the investigation of obese individuals with suboptimal levels of serum zinc, copper, and magnesium was essential for disease diagnosis. Delays in diagnosis for copper deficiency result in disability of residual neurological in patient (Kumar et al., 2016, Rios-Lugo et al., 2020, Zocchi et al., 2021). According to these studies, copper as well as zinc and magnesium has an essential role in lipid metabolism (Herman et al., 2016). Serum copper level is lower in obese subjects than healthy subjects based on previous studies (Sánchez Córdoba et al., 2016, Yang et al., 2019, Mohammad, 2020).

Some studies have reported that zinc, copper and magnesium have antioxidant properties. Also, both zinc and copper are cofactor for metalloenzymes and antioxidant enzymes (Tam et al., 2003, Mohajer et al., 2014, Mohammad, 2020).

Curcumin is a polyphenolic compound and a natural yellow pigment, also called turmeric (Safarian et al., 2019). Turmeric has the most active natural biological properties include anti-inflammatory, antioxidant, antimicrobial, anti-cancer, anti-diabetic, anti-microbial, fat modifier, anti-arthritis, analgesic, anti-ischemic and anti-depressant activities (Aggarwal and Harikumar, 2009; Gupta, et al., 2013, Trujillo et al., 2013, Mohajer et al., 2014). Curcumin also exerts antioxidant activities through various mechanisms such as inhibition and elimination of reactive oxygen species (ROS) and modulation of enzymatic antioxidants and non-enzymatic ones (Ak and Gülçin, 2008; Sahebkar et al., 2013; Panahi et al., 2014). The US Food and Drug Administration (FDA) stated the limits of safe consumption levels to a maximum of 20 mg (Cardoso et al., 2020). Despite its medicinal properties, curcumin is chemically unstable, so researchers are now looking for a suitable formulation to improve its bioavailability (Sharma, et al., 2007, Cardoso et al., 2020).

Regarding the association of curcumin with oxidative stress, the study aims to assess the effect of curcumin on zinc, magnesium and copper serum levels in obese individuals.

## Materials and Methods

**Study Design**

This study was a randomized clinical trial approved by the Mashhad University of Medical Sciences (ID: 960443) (IRCT2013082914521N1) (Mohammadi, Sahebkar et al. 2013). Thirty obese individuals aged 18 to 65 years who referred to Nutrition Department of Ghaem Hospital, Mashhad, Iran, were recruited from August 2010 to August 2012. All individuals signed written informed consent. The Exclusion criteria were breast feeding, pregnancy, suffering from systemic diseases. Obesity was defined as a BMI>30 kg/m^2^. Anthropometric indices were measured using standard protocols for all participants and they completed a questionnaire including demographic information.

**Clinical trial**

Computer randomization was used to make random distribution to groups. To implement the random allocation sequence, we used sequentially numbered sealed envelopes that was opened by someone not involved in the project. After assignment to intervention, the participants, clinical research staff and statistician were blinded. A non-researcher coded the capsules containers as A and B and this stayed confidential until data analysis. 

Curcumin capsules included 5 mg piperine plus 500 mg curcuminoids, were used (Sami Labs LTD, Bangalore, India) (Hewlings and Kalman 2017). The placebo capsules containing 5 mg of piperine were similar in shape and color to the curcumin ones. All participants received curcumin or placebo 1 g/day for a period of 30 days (n=15 in each study group). Then, the subjects were crossed over to the alternative regimen after a two-week wash-out period. In other words, there are two general phases in this project (before and after the wash-out period), in which the two groups participate including “Group 1: The patients received curcuminoids and then crossed over to the placebo; and Group 2: The patients received placebo and then crossed over to the curcuminoids.”

**Serum biochemical variables**

Blood samples were taken four times from every individual after 12-hr fasting at baseline and after a period of 30 days consumption at two general phases; before and after the wash-out period. Serum magnesium, zinc and copper levels were assayed using commercial kits by auto analyzer (model BT3000, Biotech-nica Instruments, Rome, Italy).

**Statistical analysis**

The power value of the study was 0.72 as measured using R software version 4.0.3. The Mann-Whitney U test and t-test were used for non-normally and normally dispersed factors, respectively. The period, treatment and carry over effects were evaluated for 2X2 cross-over study. A two-sided p-value of <0.05 was considered significant.

**Figure 1:**
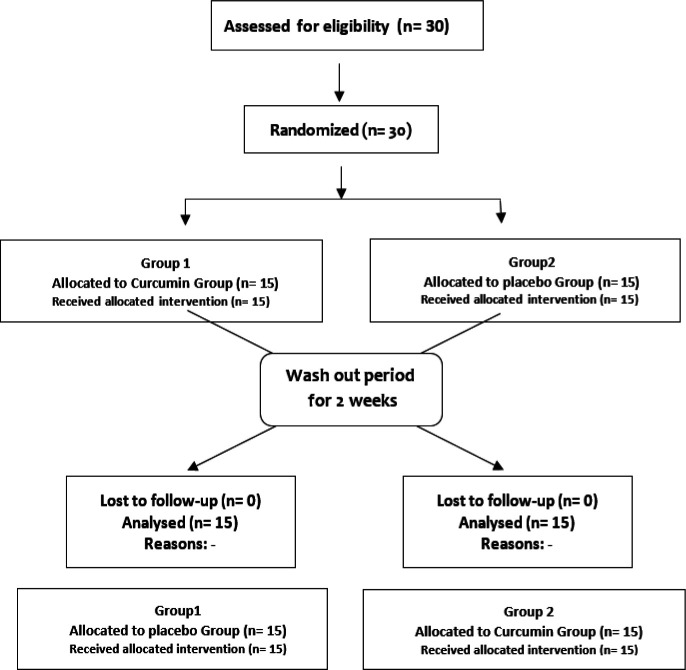
Flowchart of the study, "Phase I" and "Phase II". Group 1: The patients received curcuminoids and then crossed over to the placebo; and Group 2: The patients received placebo and then crossed over to the curcuminoids.

## Results

As Table 1 shows, the mean age of the study population was 38.37±11.51 years old and among these, 58.6 and 55.2% of women were included in the curcumin and placebo groups, respectively. The baseline clinical characteristics of the subjects are shown in Table1. Also, The normal range for lipid profile (LDL (low-density lipoproteins), HDL (high-density lipoprotein), TG (triglycerides), and TC (total cholesterol)) (Ghazizadeh et al., 2021) and Zn, Mg and Cu (Ghazizadeh et al., 2021) was defined according to previous studies. There were no significant differences in baseline features between the two groups (p>0.05). In addition, there were no significant effects (p>0.05) of curcumin supplementation at a dose of 1g/day for a period of 30 days on serum Cu, Zn, and Mg concentrations (Table 2). Figure 1 shows the study flowchart.

**Table 1:** Baseline biochemical factors in the study population

		**Phase I**			**Phase II**		
	**Normal Range **	**Group 1**	**Group 2**	**p-value**	**Group 1**	**Group 2**	**p-value**
**Women %**	-	58.6	55.2	0.791	58.6	55.2	0.791
**Men %**	-	41.4	44.8		41.4	44.8	
**Age, years**	-	38.84±11.12	37.81±12.31	0.09	-	-	-
**BMI, kg/m** ^2^	20.2-26.2	33.67±3.71	31.82±3.42	0.37	33.35±4.80	31.17±3.29	0.16
**TG, mg/dl**	26.7-225.6	102.55±29.01	126.2±57.15	0.18	100.39±47.20	120.73±64.25	0.3
**LDL-C, mg/dl**	55.6-171	119.78±23.15	118.75±27.72	0.9	119.83±33.58	124.6±27.89	0.66
**TC, mg/dl**	121.8-242.7	192.78±29.97	190.67±27.68	0.67	191.28±41.96	202.8±27.15	0.37
**HDL-C, mg/dl**	30.1-67	46.89±9.55	46.12±7.77	0.8	48.33±8.06	53.06±9.27	0.13
**Serum Zn, µg/dl**	68.6-135	106.25±29.29	94.71±30.26	0.49	102.11±26.72	91.67±18.39	0.32
**Serum Cu, µg/dl**	30-181.65	117.17±26.51	112.12±28.62	0.74	120.71±37.4	110.23±34.95	0.48
**Serum Zn/Cu**	-	0.93±0.31	0.89±0.36	0.8	0.92±0.34	0.92±0.29	0.98
**Serum Mg, mg/dl**	0.82-1.23	2.93±1.68	2.15±0.81	0.33	2.05±0.67	2.25±0.85	0.53

Data are presented as Mean±SD or interquartile range. Phase I: before wash-out period, Phase II: after wash-out period. Group 1: the patients received curcuminoids and then crossed over to the placebo; Group 2: the patients received placebo and then crossed over to the curcuminoids.BMI: Body Mass Index, TG: Triglyceride, TC: Cholesterol, LDL-C: Low-density lipoprotein, HDL-C: High-density lipoprotein, Zn: Zinc, Cu: Copper, Zn/Cu: Zinc ratio copper

**Table 2:** Curcumin’s effects on serum levels of Cu, Zn and Mg in the study population

		**N**	**Phase I**		**Phase II**		**p-value**	
**Variable**	**Study group**		Pre treatment	Post treatment	Pre treatment	Post treatment	Treatment effect	Period effect
**Serum Zn, µg/dl**	**Group 1**	15	106.25(29.29)	86.17(20.35)	102.11(26.72)	90.69(27.85)	0.75	0.99
	**Group 2**	15	94.71(30.26)	95.16(30.74)	91.67(18.39)	112.07(24.18)		
**Serum Cu, µg/dl**	**Group 1**	15	117.17(26.51)	130.27(38.21)	120.71(37.39)	123.95(27.16)	0.97	0.93
	**Group 2**	15	112.12(28.62)	116.10(58.53)	110.23(34.95)	122.5(39.75)		
**Serum Zn/Cu**	**Group 1**	15	0.93(0.31)	0.71(0.24)	0.92(0.34)	0.74(0.19)	0.97	0.9
	**Group 2**	15	0.89(0.36)	1.01(0.51)	0.92(0.29)	1.04(0.42)		
**Serum Mg, mg/dl**	**Group 1**	15	2.93(1.68)	2.09(0.81)	2.05(0.67)	1.93(0.62)	0.7	0.85
	**Group 2**	15	2.15(0.81)	2.12(1.10)	2.25(0.85)	2.33(0.86)		

Phase I: Before wash-out period, Phase II: After wash-out period. Group 1: The patients received curcuminoids and then crossed over to the placebo; Group 2: The patients received placebo and then crossed over to the curcuminoids. Zn: Zinc, Cu: Copper, Zn/Cu: Zinc ratio copper

## Discussion

This randomized double-blind crossover trial study has been the first trial one that investigated the effect of curcumin on serum Zn, Cu and Mg levels in obese people. Today, obesity is considered a serious threat to health. Studies have shown a negative relationship between serum Mg and Zn levels and obesity (Shamnani, et al., 2018; Gu et al.,2019, Rios-Lugo et al., 2020). A meta-analysis study suggested that a higher serum copper concentration may be associated with obesity in children and adults (Gu et al., 2020). Copper and zinc as two essential trace elements play an important role in biological processes so that they may have a significant impact on the pathogenesis of metabolic diseases. In a study, zinc and copper deficiency were associated with a higher risk of CVDs and diabetes, and an imbalance between Zn and Cu levels led to oxidative stress and insulin resistance (Hamasaki et al., 2016). Darroudi et al., demonstrated that serum zinc and copper were altered in Iranian adults who were metabolically obese but in normal weight. They suggested that copper and zinc were the strong risk factor for metabolic syndrome (MetS) in normal weight individuals (Darroudi et al., 2019) .

Curcumin is able to exert antioxidant effects by reducing oxygen and nitrogen free radicals or modulating the cellular defense system (Li et al., 2015). Also, curcumin anti-inflammatory and antioxidant properties can prevent the progression of inflammatory reactions (Panahi et al., 2018). Obesity is considered a low-grade chronic metabolic inflammation, and empirical evidence has shown that curcumin is effective in reducing the incidence of obesity-related diseases (Bradford, 2013).

There is little evidence about the effect of curcumin on trace element levels in obese individuals. According to the results of the present study, consumption of 500 mg of curcumin twice a day, with the elimination of confounders, had no significant effect on magnesium, zinc, copper, and zinc to copper ratio in obese people. Consistent to our results, a study on 30 obese patients showed that a daily intake of 1 g/day of curcumin together with black pepper at a dose of 10 g/day for four weeks did not have a significant effect on copper and zinc, however, it was associated with a significant increase in zinc/copper and decrease in copper to zinc ratio (Mohajer et al., 2014). In a double-blind clinical trial on 120 individuals with metabolic syndrome, it was shown that after daily consumption of 1 g of curcumin and phospholipidated curcumin for 6 weeks, Zn concentration and Zn/Cu levels were significant and Zn was increased so that in the phospholipidated curcumin group, it was higher, but there was no significant effect on Cu (Safarian et al., 2019). We have previously reported that phospholipidated curcuminoids (1 g per day), for a period of 6 weeks, does not have effects on serum cholesteryl ester transfer protein level (Javandoost et al., 2018), or sleep-duration (Saberi-Karimian et al., 2021) in subjects with MetS.

The difference between our study and other studies can be due to the dose and duration of curcumin intake in obese people. According to studies, doses above 12 g per day are safe and tolerable for humans and only mild side effects have been reported (Manjunatha and Srinivasan, 2006; Hsu and Cheng, 2007). However, in this study, a dose of 1 g per day was investigated.

In this study, we investigated the effect of curcumin on serum Cu, Zn and Mg levels in obese people. Our results demonstrated that curcumin did not affect serum Cu, Zn, Mg concentrations in obese subjects. Since, the evidence about the effect of curcumin on trace elements concentrations in obese individuals is little, it is suggested to investigate the association of these factors in future studies.

## Conflicts of interest

The authors have declared that there is no conflict of interest.
